# Prediction of coronary disease incidence by biomarkers of inflammation, oxidation, and metabolism

**DOI:** 10.1038/s41598-018-21482-y

**Published:** 2018-02-16

**Authors:** Isaac Subirana, Montserrat Fitó, Oscar Diaz, Joan Vila, Albert Francés, Eva Delpon, Juan Sanchis, Roberto Elosua, Daniel Muñoz-Aguayo, Irene R. Dégano, Jaume Marrugat

**Affiliations:** 1 CIBER of Epidemiology and Public Health (CIBERESP), Instituto de Salud Carlos III (ISCIII), Madrid, Spain; 2 Cardiovascular Epidemiology and Genetics Research Group, Program of Epidemiology and Public Health, Hospital del Mar Medical Research Institute (IMIM), Barcelona, Spain; 3 Cardiovascular Risk and Nutrition Research Group, Program of Epidemiology and Public Health, IMIM, Barcelona, Spain; 4 CIBER of Physiopathology of Obesity and Nutrition (CIBEROBN), ISCIII, Madrid, Spain; 5 Hospital del Mar, Barcelona, Spain; 6 Universidad Complutense, Madrid, Spain; 7 Servei de Cardiologia, INCLIVA, Departament de Medicina, Hospital Clínico Universitario de Valencia, Universitat de Valencia, Valencia, Spain; 8 CIBER of Cardiovascular Diseases (CIBERCV), ISCIII, Madrid, Spain; 9 REGICOR Group, Program of Epidemiology and Public Health, Hospital del Mar Medical Research Institute (IMIM), Barcelona, Spain

## Abstract

The effect of circulating biomarkers in predicting coronary artery disease (CAD) is not fully elucidated. This study aimed to determine the relationship with CAD and the predictive capacity of nine biomarkers of inflammation (TNF-α, IL-10, IL-6, MCP-1, CRP), oxidation (GHS-Px), and metabolism (adiponectin, leptin, and insulin). This was a case-cohort study, within the REGICOR population-cohorts (North-Eastern Spain), of 105 CAD cases and 638 individuals randomly selected from a cohort of 5,404 participants aged 35–74 years (mean follow-up = 6.1 years). Biomarkers’ hazard ratio (HR)/standard deviation was estimated with Cox models adjusted for age, sex, and classical risk factors. Discrimination improvement and reclassification were analyzed with the c-index and the Net reclassification index (NRI). GHS-Px (adjusted HRs = 0.77; 95%CI:0.60–0.99), insulin (1.46; 1.08–1.98), leptin (1.40; 1.03–1.90), IL-6 (1.34; 1.03–1.74), and TNF-α (1.80; 1.26–2.57) were significantly associated with CAD incidence. In the model adjusted for all biomarkers, TNF-α (1.87;1.31–2.66) and insulin (1.59;1.16–2.19) were independently associated with CAD. This final model, compared to a model without biomarkers, showed a c-index difference of 1.3% (−0.7, 3.2) and a continuous NRI of 33.7% (2.6, 61.9). TNF-α and insulin are independently associated with CAD incidence and they improve reclassification when added to a model including classical risk factors.

## Introduction

More than 30,000 proteins circulate in human plasma, mostly albumin and immunoglobulins. The remaining proteins are present at varying concentrations (e.g., micromolar [10^−6^ M] such as adiponectin, nanomolar [10^−9^ M] such as leptin, or picomolar [10^−12^ M] such as interferon gamma) but most in even lower amounts^1^. Plasma concentrations of some of these proteins might be good candidates to predict development of coronary artery disease (CAD), particularly inflammatory^2,3^, metabolic^4^, and oxidative^5^ biomarkers closely related to atherosclerosis.

Chronic inflammation in cardiovascular disease (CVD) appears to be associated with the oxidative/anti-oxidative homeostasis, yielding an accumulation of oxidized low-density lipoproteins (LDL) in the arterial wall^5^. Endogenous antioxidant enzymes such as glutathione peroxidase (GHS-Px)^6^ play a major role in maintaining oxidative homeostasis, acting as the first-line defense against free radicals. The oxidative process perpetuates an inflammatory response in the subendothelial space, as activated cells secrete pro-inflammatory molecules. Expression of tumor necrosis factor alpha (TNF-α)^7^ and interleukin (IL)-1 by endothelial cells and monocytes triggers a response involving monocyte chemo-attractant protein-1 (MCP-1), which promotes monocyte recruitment, macrophage activation, and signal expression^6,8^. Other cytokines, among them IL-10 and IL-6, are released in the process of atherosclerosis development^9–11^, and C-reactive protein (CRP) synthesis is increased by the liver. CRP activates the complement system that promotes phagocytosis which allows clearance of necrotic tissues in the atherosclerotic plaque and perpetuates the inflammatory response^3,7^.

Within this inflammatory response, metabolism biomarkers–e.g., insulin, leptin, and adiponectin–also play an important role. Adiponectin correlates with lower risk of CAD events, probably through inhibiting TNF-α action in endothelial cells, while increased leptin concentration is associated with CVD^4,12,13^.

Most of the aforementioned biomarkers have been consistently, but usually separately, found to be associated with future development of CAD events^14,15^. The measurement of a combination of biomarkers mutually adjusted may offer additive predictive information and improve cardiovascular risk stratification^16^. However, researchers have explored only some of the potential biomarkers to determine their combined effect on the CAD predictive capacity^17^.

The objectives of the present study were: (1) to determine the individual and mutually adjusted relationship between the development of CAD events and systemic levels of a set of biomarkers of inflammation, oxidation, and metabolism and (2) to test the biomarkers’ incremental predictive capacity for CAD, beyond that of classical cardiovascular risk factors.

To analyze these objectives we used the REGICOR population cohorts^18^ from the Girona province in North-Eastern Spain. The individuals from these cohorts were recruited in 1995–2000–2005 and have been extensively characterized regarding cardiovascular and lifestyle risk factors and incidence of cardiovascular events.

## Results

### Baseline characteristics

The randomly selected subcohort and the cases included 667 and 117 individuals, respectively. There were 13 individuals that were in both groups. We finally included the 105 cases and 638 subcohort members that had sufficient biological sample to complete the laboratory tests (Supplementary Fig. S1).

Cases were older and more frequently men than individuals of the subcohort (Table 1). Cases had a worse profile of CV risk factors except for estimated glomerular filtration rate (eGFR). Biomarkers of inflammation, oxidation, and metabolism differed between cases and subcohort participants except for leptin, IL-10, and MCP-1 (Table 2). Compared to the subcohort, cases showed a higher concentration of hs-CRP, insulin, IL-6, and TNF-α, and a lower concentration of GHS-Px, and adiponectin.

**Table 1:** Baseline characteristics of patients with coronary artery disease events (cases) and of the subcohort without events.

	Subcohort	CAD Cases	*p* value
	(n = 638)	(n = 105)	
Age (mean (sd))	53.8 (10.6)	61.1 (9.72)	<0.001
Sex, men (n (%))	295 (46.2%)	72 (68.6%)	<0.001
Current smoker (n (%))	134 (21.4%)	40 (38.5%)	<0.001
Diabetes (n (%))	84 (13.2%)	33 (31.4%)	<0.001
Glycemia, mg/dL (median [IQR])	93.0 [86.0, 103]	100 [90.0, 125]	<0.001
Hypertension, treated (n (%))	103 (16.6%)	39 (37.5%)	<0.001
Diastolic blood pressure, mmHg (mean (sd))	79.2 (9.68)	82.3 (11.5)	0.004
Systolic blood pressure, mmHg (mean (sd))	126 (18.1)	137 (20.1)	<0.001
Hypercholesterolemia, treated (n (%))	81 (12.7%)	25 (23.8%)	0.004
Total cholesterol mg/dL (mean (sd))	212 (43.5)	227 (42.1)	0.001
HDL cholesterol mg/dL (mean (sd))	53.1 (13.2)	45.9 (15.1)	<0.001
LDL cholesterol mg/dL(mean (sd))	138 (38.9)	150 (37.9)	0.007
Triglycerides mg/dL (median [IQR])	93.0 [67.0, 128]	123 [94.0, 190]	<0.001
Body mass index(mean (sd))	27.3 (4.64)	28.6 (4.73)	0.020
eGFR (mL/min) (median [IQR])	83.1 [73.6, 93.5]	85.5 [74.1, 97.2]	0.930

Medians, interquartile ranges, means and standard deviations were obtained without variable transformation. *p* values were obtained taking into account the case-cohort design and with log-transformed glucose, triglycerides and eGFR. CAD, coronary artery disease; eGFR, estimated glomerular filtrate rate; HDL, high-density lipoprotein; IQR, interquartile range; LDL, low-density lipoprotein; sd, standard deviation.

**Table 2:** Biomarker association with coronary artery disease in cases and subcohort individuals.

	Subcohort	CAD Cases	*p* value
	(n = 638)	(n = 105)	
hs-CRP mg/dL (median [IQR])	0.12 [0.04, 0.32]	0.22 [0.07, 0.59]	0.001
GHS-Px U/L (mean (sd))	718 (102)	690 (110)	0.011
Adiponectin µg/mL (median [IQR])	5.04 [3.28, 8.30]	3.91 [2.73, 6.98]	0.009
Insulin pg/mL (median [IQR])	216 [119, 349]	332 [207, 578]	<0.001
Leptin ng/mL (median [IQR])	6.44 [3.18, 12.2]	6.94 [4.16, 11.6]	0.119
Interleukin-6 pg/mL (median [IQR])	1.59 [1.06, 2.55]	2.42 [1.70, 3.87]	<0.001
TNF-α pg/mL (median [IQR])	0.80 [0.32, 1.57]	1.34 [0.82, 1.94]	<0.001
Interleukin-10 pg/mL (median [IQR])	0.37 [0.17, 0.67]	0.32 [0.17, 0.50]	0.342
MCP-1 pg/mL (median [IQR])	331 [249, 414]	360 [277, 440]	0.061

Medians, interquartile ranges, means and standard deviations were obtained without variable transformation. *p* values were obtained taking into account the case-cohort design and all variables were log-transformed except for GHS-Px. CAD, coronary artery disease; GHS-Px, Glutathione peroxidase; hs-CRP, high sensitivity C-reactive protein; IQR, interquartile range; MPC-1, Monocyte chemoattractant protein-1; sd, standard deviation; TNF-α, Tumor necrosis factor alpha.

### Correlation of biomarkers and association with CAD events

The Pearson correlation and the variation inflation factor (VIF) are shown in Supplementary Table S1. None had a VIF > 2.5, which indicates that no excessive multiple linear correlation existed between any single biomarker and the rest.

The adjusted effect of one standard deviation of each biomarker on CAD incidence is presented in Table 3. GHS-Px, insulin, leptin, IL-6, and TNF-α, were associated with CAD events even after adjustment for classical CV risk factors and statin use. GHS-Px showed a protective effect, while insulin, leptin, IL-6, and TNF-α, increased the risk of CAD events. With minimal adjustment for age and sex adiponectin had a protective effect for CAD events. Hs-CRP, IL-10, and MCP-1 were not associated with CAD events in any of the models.

**Table 3:** Hazard ratios [and 95% confidence intervals] of coronary artery disease incidence for one standard deviation of the considered biomarkers after adjustment for classical risk factors.

	Adjusted for age and sex	Adjusted for age, sex, & risk factors^b^	Adjusted for age, sex, and risk factors^b^ & statin use
Hs-CRP^a^	1.26 [0.99, 1.60]	1.00 [0.76, 1.33]	1.00 [0.76, 1.33]
GHS-Px	0.78 [0.62, 0.98]	0.77 [0.60, 0.99]	0.77 [0.60, 0.99]
Adiponectin^a^	0.76 [0.59, 0.99]	0.93 [0.68, 1.27]	0.92 [0.67, 1.26]
Insulin^a^	1.84 [1.40, 2.43]	1.47 [1.08, 2.01]	1.46 [1.08, 1.98]
Leptin^a^	1.56 [1.18, 2.07]	1.40 [1.03, 1.90]	1.40 [1.03, 1.90]
Interleukin-6^a^	1.53 [1.21, 1.94]	1.34 [1.03, 1.75]	1.34 [1.03, 1.74]
TNF-α^a^	2.05 [1.47, 2.86]	1.80 [1.26, 2.56]	1.80 [1.26, 2.57]
Interleukin-10^a^	0.91 [0.73, 1.14]	0.93 [0.73, 1.20]	0.94 [0.73, 1.20]
MCP-1^a^	1.06 [0.84, 1.33]	1.03 [0.80, 1.32]	1.03 [0.80, 1.32]

^a^Log-transformed; ^b^Systolic blood pressure, diastolic blood pressure, high-density lipoprotein-cholesterol, total cholesterol, diabetes, and smoking. GHS-Px, Glutathione peroxidase; hs-CRP, high sensitivity C-reactive protein; MPC-1, Monocyte chemoattractant protein-1; TNF-α: tumor necrosis factor alpha.

### Effect of biomarkers on CAD prediction

After a sequential elimination of the non-significant biomarkers, insulin, and TNF-α were significantly and independently associated with CAD events in all models (Table 4). IL-6 was associated with CAD events only in the model adjusted for age and sex. In this model, the inclusion of IL-6, insulin, and TNF-α significantly improved discrimination and reclassification (both continuous and categorical NRI)(Table 4, column 1). In the models adjusted for the rest of CV risk factors and statin use, the inclusion of insulin, and TNF-α did not improve discrimination significantly but increased reclassification when measured with the continuous NRI (Table 4, columns 2 and 3).

**Table 4:** Mutually adjusted hazard ratios [and 95% confidence intervals] of coronary artery disease incidence, reclassification and discrimination.

	Adjusted for age and sex	Adjusted for age, sex, & risk factor^e^	Adjusted for age, sex, risk factor^e^ & statin use
Interleukin-6^a^	1.35 [1.03, 1.75]	—	—
TNF-α^a^	2.02 [1.46, 2.81]	1.88 [1.32, 2.68]	1.87 [1.31, 2.66]
Insulin^a^	1.90 [1.41, 2.58]	1.62 [1.17, 2.25]	1.59 [1.16, 2.19]
Reclassification			
Continuous NRI (%)	73.0 [48.1, 95.7]	43.3 [14.5, 69.3]	33.7 [2.6, 61.9]
Categorical NRI (%)^b^	23.5 [11.4, 35.6]	5.2 [−5.4, 15.8]	6.8 [−4.0, 17.6]
Cases	22.8 [11.7, 34.0]	4.2 [−5.4, 13.8]	6.5 [−3.3, 16.2]
Subcohort	0.7 [−2.4, 3.8]	1.0 [−1.6, 3.6]	0.3 [−2.4, 3.1]
Clinical NRI^c^	36.6 [3.7, 69.4]	9.9 [−18.1, 38.0]	11.7 [−17.2, 40.7]
Cases	38.8 [9.7, 67.8]	7.7 [−10.3, 25.7]	12.8 [−5.9, 31.6]
Subcohort	−2.2 [−14.0, 9.6]	2.2 [−14.1, 18.6]	−1.1 [−19.3, 17.1]
Discrimination (C-index)			
Without biomarkers^d^	74.3 [69.3, 79.4]	81.3 [77.2, 85.5]	81.3[78.1, 84.6]
With biomarkers^e^	79.3 [74.7, 84.0]	82.5 [78.6, 86.4]	82.6 [79.3, 85.9]
Difference	5.0 [1.6, 8.4]	1.1 [−1.0, 3.3]	1.3 [−0.7, 3.2]

^a^Log-transformed. ^b^For the categorical NRI, 5% and 10% were used as cutoff points; ^c^Reclassification of intermediate risk (5% to 10% risk) participants. ^d^Adjusted for risk factors described in the column heading alone. ^e^Adjusted for risk factors described in the column heading, and the significant biomarkers. ^e^Systolic blood pressure, diastolic blood pressure, high-density lipoprotein-cholesterol, total cholesterol, diabetes, and smoking. CPE, concordance probability estimate; NRI, net reclassification index; TNF-α, tumor necrosis factor alpha.

## Discussion

Baseline values of systemic GHS-Px, IL-6, insulin, leptin, and TNF-α were associated with 6-year incidence of CAD, independently of classical risk factors; this was not true for other well-known biomarkers (hs-CRP, IL-10, adiponectin, and MCP-1). Only insulin and TNF-α were independently associated with CAD incidence when considered together in fully adjusted models. These two biomarkers improved reclassification measured with the continuous NRI in the model adjusted for classical risk factors and statin use.

Among all biomarkers, inflammatory ones seem the most promising for the assessment of CVD risk in the general population^19^. After adjustment for CV risk factors and statin use we found a significant association between CAD events and systemic levels of GHS-Px, IL-6, insulin, leptin, and TNF-α. Our study confirms, in a south European population, the association of CAD events with GHS-Px, IL-6, and TNF-α described in previous studies^20–23^. A recent case-cohort and meta-analysis yielded similar individual HR per standard deviation in adjusted models for IL-6 (1.26 *vs* 1.34) but lower for TNF-α (1.14 *vs* 1.80) compared with our results^14^. These difference could be due to the longer median follow-up and the older participants included in their study. We also found a direct association between leptin levels and CAD events while recent meta-analyses have shown significant associations in minimal adjusted models but non-significant associations in models adjusted for CV risk factors^24,25^. This discrepancy is probably due to the lower number of cases included in our study. In our study insulin was directly associated with CAD events. Recent analyses have shown that insulin markers may be strongly associated with CAD, particularly proinsulin levels^26^.

Our results also showed non-significant associations of hs-CRP, IL-10, adiponectin, and MCP-1 with CAD in fully adjusted models. Hs-CRP has consistently predicted CVD in large prospective studies^16,27,28^, and a HR per standard deviation of 1.23 has been reported^16^, very similar to the 1.26 found in the present study. However, due to evidence of reporting bias the true association of Hs-CRP with future CAD events is not clear and, in accordance with our results, its contribution to CVD risk assessment is assumed to be small^29^. IL-10 has been associated with CVD events in elderly individuals^30^ but there are no consistent data of its association with CAD events in general population. Neither adiponectin nor MCP-1 were strong predictors of coronary events in healthy individuals at intermediate risk in addition to CV risk factors^31^.

Despite promising results in some studies^16,21,32,33^, efforts to systematically analyze risk prediction improvements using a set of atherosclerosis-related biomarkers have been scarce. The relationship between some of the tested biomarkers and CVD or CAD has been reported in a limited number of articles. These studies analyzed a large set of biomarkers^21,22,34^, a subset of the assayed biomarkers^35^ or a biomarker score^16,25,34,36^. The studies analyzing a large set or a subset of a biomarker set found an improvement in discrimination and reclassification. Such as the work by Herder *et al*., in which the addition of 13 inflammatory biomarkers, including CRP, IL-6, MCP-1, adiponectin, and leptin, among others, yielded a modest but significant improvement of coronary risk prediction in adjusted models^21^. On the other hand, the effect of a biomarker score differed between studies. Blankenberg and collaborators showed no improvement of risk estimation by single biomarkers but significant improvement of discrimination and reclassification by a biomarker score including CRP, N-terminal pro-B-type natriuretic peptide, and troponin I^16^. The inclusion of these independent biomarkers also improved 10-year CVD risk prediction in a larger cohort^32^. In another study, the addition of a biomarker score including CRP and IL-6 among others, did not improve reclassification^35^. In our study, the addition of insulin and TNF-α showed an improvement in reclassification but not in discrimination as in the study by Würtz *et al*.^36^.

While IL-6, insulin, and TNF-α were independently associated with CAD incidence when their effect was adjusted for age and sex, a significant independent effect persisted only for insulin and TNF-α in the fully adjusted model. These results suggest that high levels of insulin, and TNF-α may indicate the presence of coronary artery atherosclerosis that translates into CAD events during a 6-year follow-up. In addition, insulin levels were associated with 6-year CAD incidence independently of adiponectin and leptin, and of inflammatory cytokines.

Our results are in line with recent European guidelines for CVD prevention, which state that the known circulating biomarkers have limited value for CVD risk assessment^29^. However, the lack of discrimination improvement and the limited change in reclassification could also be due to the absence of recently identified biomarkers which would be more strongly associated with CAD, such as kallikrein, lipoprotein a, and matrix metalloproteinase 9^33^. It is also possible that a multimodal strategy combining circulating biomarkers and vascular markers such as coronary artery calcium could yield larger improvements in CVD risk assessment^34,37^.

The present study has several strengths. First, we included a large number of biomarkers of inflammation, oxidation, and metabolism –three different but complementary pathways of atherosclerotic disease-. Second, we used the well characterized REGICOR population cohorts which have high quality data and have previously been used to examine CAD risk prediction. These cohorts are representative of a population of approximately 600,000 individuals supporting the external validity of the results. Third, samples were analyzed in a unique laboratory to ensure reliable determinations of biomarkers^18^. Fourth, we used a robust design that allowed the calculation of discrimination and reclassification statistics.

Our study has also limitations that should be considered. First, no basal biological sample was available for 12 of 117 cases and for 29 of 667 individuals of the subcohort. These losses represent an approximately similar proportion of cases and subcohort candidates, indicating that sample availability was not associated with case status. Second, the cost of assessing the full set of biomarkers under assay precluded testing in the full cohort. Therefore, the statistical power is limited to HR >1.58. Finally, it is possible that longer follow-up could slightly modify our results.

In conclusion, our study showed an association of systemic levels of GHS-Px, IL-6, TNF-α, insulin, and leptin, with incidence of CAD after adjustment for CV risk factors and statin use. The combination of TNF-α and insulin was associated with CAD incidence independently of risk factors and biomarkers. However, in fully adjusted models, the inclusion of TNF-α and insulin achieved only marginal improvement in reclassification and no improvement in discrimination compared to classical risk factors.

## Methods

This study was approved by the *Parc de Salut Mar* Ethics Committee (#2011/4309/I) and was performed in accordance with the Declaration of Helsinki. All participants signed a written informed consent.

### Participants and design

We included individuals from the 2005 REGICOR (*Registre Gironí del Cor*) population survey of 6,352 participants, from the Girona area in Spain^18^. Inclusion and follow-up was carried out in 2004–2006 and in 2009–2011, respectively. For this study, participants aged 35–74 years and with no cardiovascular disease (CVD) at baseline were included (n = 5,404). A case-cohort study was designed with all cases during follow-up (n = 117) and a random subsample of the cohort (subcohort) (n = 667). Only participants with sufficient basal samples were included (105 and 638, respectively).

### Power calculation

With a sample size of 743, an outcome incidence of 2.7% in the participants without previous CVD, and assuming a 5% type I error, we had 80% statistical power to identify a hazard ratio (HR) ≥ 1.58 per standard deviation of a normally distributed biomarker. Power calculation was based on “ccsize” function from the “gap” R package.

### Follow-up and composite endpoint

Participants were followed-up by re-examination and a structured telephone interview. The composite endpoint included fatal or nonfatal first occurrence of myocardial infarction or angina (International Classification of Diseases (ICD)-9 codes: 410, 411.0, 411.1, 412, 414, 429; and ICD-10 codes: I21-I25, including subtypes). Nonfatal events were validated with medical records. The participant database was linked with the Catalan Death Registry and the Mortality Log of the Spanish Ministry of Health to identify fatal cases (ICD-9 codes: 410–414; and ICD-10 codes: I20-I22, I24, I25). Diagnoses were collected from autopsies if performed and from medical records. All events were classified by an expert committee according to standardized criteria: myocardial infarction was defined according to the American Heart Association definition for epidemiology and clinical research studies; angina was defined according to the presence of symptoms and objective demonstration of ischemia or presence of coronary stenosis; death due to CAD was determined by the reported ICD codes.

### Laboratory determinations

Blood samples were obtained in the morning after 10–14 hours fasting, centrifuged, aliquoted, and frozen at −80 °C until assayed. Basal serum glucose, total cholesterol, and triglycerides, were determined by enzymatic methods, and high-density lipoprotein cholesterol (HDL) by a direct methodology (Roche Diagnostics, Basel, Switzerland), in a Cobas Mira Plus autoanalyzer (Roche Diagnostics, Basel, Switzerland). High-sensitivity CRP (hs-CRP) and creatinine were determined in plasma by immunoturbidimetry (ABX Diagnostic, Montpellier, France). eGFR was obtained using the Modification of Diet in Renal Disease equation^38^. LDL cholesterol was calculated by the Friedewald equation when triglycerides were lower than 300 mg/dL.

Plasma GSH-Px activity was measured by a modification of the Paglia and Valentine method, using cumene hydroperoxide to oxidize glutathione (Ransel RS 505, Randox Laboratories, Crumlin, UK).

The simultaneous determination of adiponectin, leptin, and insulin was performed in plasma with bead-based multiplexing technology, using a XMAG-Luminex assay (Biorad, Hercules, California, USA). Standards, blanks, controls, and patients samples were applied in duplicate. The fluorescence signal was read on a BioPlex 200 equipment (Biorad). A single-plex Luminex assay was performed to determine serum MCP-1 (R&D Systems, Minneapolis, USA). Finally, plasma concentrations of TNF-α, IL-10, and IL-6 were analyzed by high-sensitivity ELISAs (R&D Systems, Minneapolis, USA). External quality assessment was performed with Quality Control BIORAD (Bio-Rad, Hercules, California, USA) and Assessment-SEQC (*Sociedad Española Química Clínica*, Barcelona, Spain).

The coefficient of variation range was 1.60%-3.29% for the general biochemistry (glucose, total and HDL cholesterol, triglycerides, and creatinine), 3.39%-7.07% for the GSH-Px activity, 1.75%-6.35% for the immunoturbidimetric assay, 10.89%-15.00% for the ELISAs, and 7.30%-14.00% for the Luminex assays.

### Other basal measurements

Systolic and diastolic blood pressure (mmHg), body mass index (BMI, kg/m^2^), smoking status (current, former, never smoker), diabetes (yes/no), hypertension history (yes/no), and hypertension and dyslipidemia treatment (yes/no) were obtained by standardized and validated methods^18^.

### Statistical analyses

Baseline characteristics were summarized as mean and standard deviation or as median and first and third quartiles if they were continuous and normally or non-normally distributed, respectively. Categorical variables were summarized by proportions.

Correlation between biomarkers was analyzed with the Pearson correlation coefficient and with the VIF, which measured multiple linear correlations between each biomarker and the rest.

The Lin-Ying weighted estimate for random sample case-cohort design was used to estimate differences in demographic, risk factors, and biomarkers, between individuals in the case and subcohort groups^39^. Cox proportional hazard regression, weighted by the Lin-Ying method, was used to model time to CAD event and estimate the HR for the effect of one standard deviation increase in biomarker levels. An incremental adjustment strategy was used to test each biomarker effect: the first model was adjusted for age and sex; the second added systolic and diastolic blood pressure, HDL and total cholesterol as continuous variables, and diabetes and smoking as categorical variables; and the third incorporated statin use as a dichotomous variable to take into account the pleiotropic effect of these drugs. In addition, for each biomarker, the effect of one standard deviation was mutually adjusted for the other biomarkers in three backward-elimination models with the same adjustment strategy as above. When necessary, the biomarkers were log-transformed to normalize their distribution.

Contribution to predictive capacity of biomarkers over classical risk factors was assessed by the discrimination improvement, computing the increment of C-statistic as described for case-cohort studies^40,41^. Reclassification was assessed with the Net Reclassification Index (NRI) both categorical and continuous. For the categorical NRI, 5% and 10% were used as cutoff points, as these correspond to the cutoff points of CHD risk at 10 years in Spain. Confidence intervals were obtained by bootstrapping.

Statistical analyses were performed using R version 3.4.0^42^. The “cch” function from the “survival” R package was used to obtain the Lin-Ying weighted estimates.

### Data Availability

The dataset analyzed during the current study is available from the corresponding authors on reasonable request.

## Electronic supplementary material

Supplementary data
