# Supplementary material for: Prediction of coronary disease incidence by biomarkers of inflammation, oxidation, and metabolism
Source: Sci Rep. 2018 Feb 16;8:3191. doi: 10.1038/s41598-018-21482-y (PMC5816603; doi:10.1038/s41598-018-21482-y)

SUPPLEMENTARY INFORMATION

Title of the manuscript: Prediction of coronary disease incidence by biomarkers of inflammation, oxidation, and metabolism

Author list: Isaac Subirana, Montserrat Fitó, Oscar Diaz, Joan Vila, Albert Francés, Eva Delpon, Juan Sanchis, Roberto Elosua, Daniel Muñoz-Aguayo; Irene R Dégano*, Jaume Marrugat*

Supplementary Table S1 online. Pearson correlation coefficients (above diagonal) between pairs of biomarkers in ordinal categories and variance inflation factor of each variable with the rest of biomarkers.

	VIF	CRP	GHS-Px	Adiponectin	Insulin	Leptin	IL-6	TNF-α	IL-10	MCP-1
**hs-CRP**	1.53	100.0	-0.02	-0.15	0.34	0.30	0.53	0.10	0.03	0.07
**GHS-Px**	1.08		100.0	0.09	-0.13	-0.14	-0.07	-0.17	-0.01	0.01
**Adiponectin**	1.52			100.0	-0.33	0.09	-0.08	0.04	0.03	-0.01
**Insulin**	1.93				100.0	0.54	0.28	0.03	-0.02	0.06
**Leptin**	1.72					100.0	0.25	0.09	0.01	0.09
**IL-6**	1.53						100.0	0.21	0.01	0.15
**TNF-α**	1.10							100.0	0.04	0.04
**IL-10**	1.01								100.0	-0.03
**MCP-1**	1.05									100.0

All variables were log-transformed except for GHS-Px.GHS-Px. Glutathione peroxidase; hs-CRP, high sensitivity C-reactive protein; IL Interleukin; MPC-1, Monocyte chemoattractant protein-1; TNF-α, tumor necrosis factor alpha; VIF, variance inflation factor.

Figure S1 online. Flowchart of the study participants. CAD coronary artery disease; MCP-1 monocyte chemoattractant protein-1; IL-10 interleukin-10; IL-6 interleukin-6; hs-CRP High-sensitivity C-reactive protein; GSH-Px glutathione peroxidase activity; TNF-α tumor necrosis factor alpha

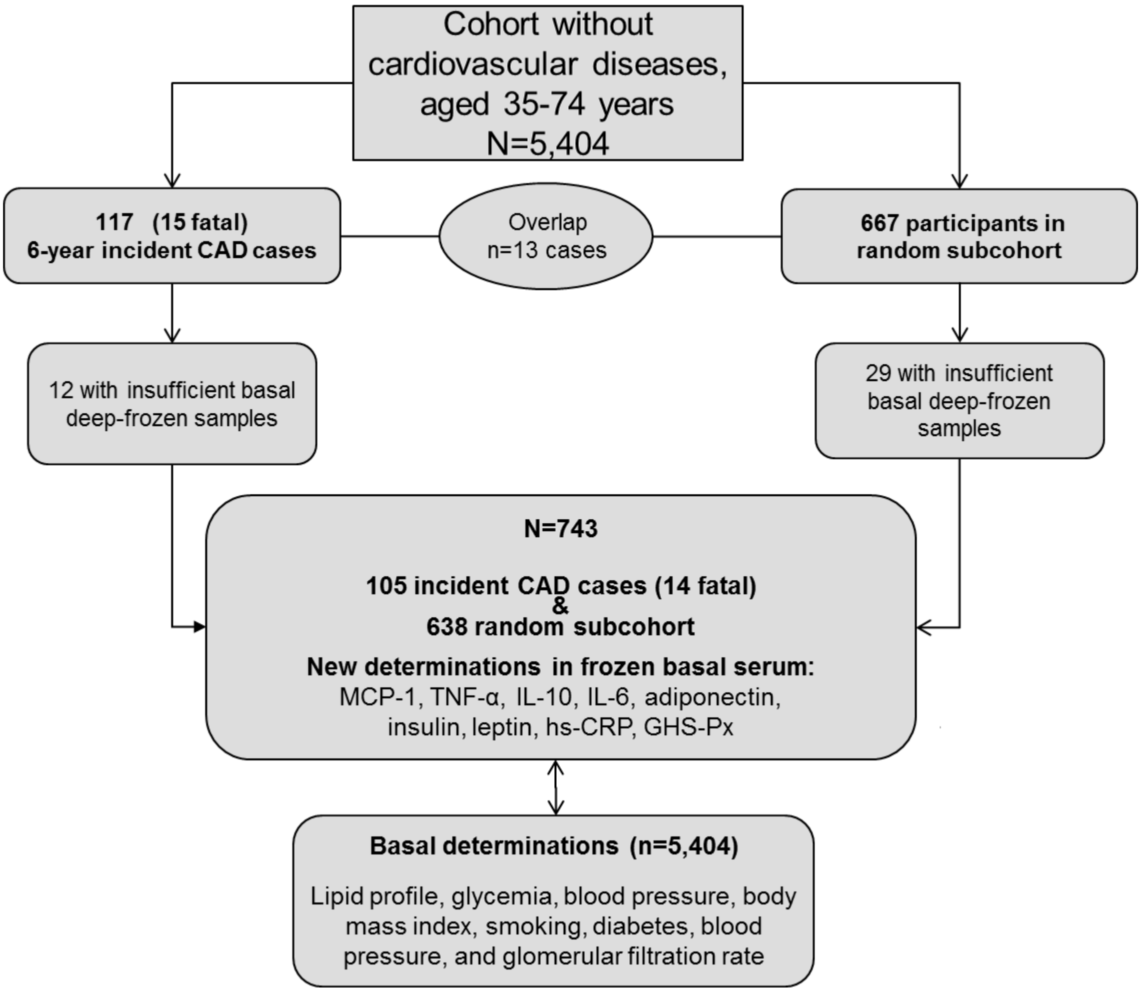

Supplement: Supplementary file 1 — Supplementary data [file 41598_2018_21482_MOESM1_ESM.pdf]
